# Influence of immigrant background on the outcome of total hip arthroplasty: better outcome in 280 native patients in Bosnia and Herzegovina than in 449 immigrants living in Sweden

**DOI:** 10.1177/11207000231182321

**Published:** 2023-10-05

**Authors:** Ferid Krupic, Slavko Manojlovic, Svemir Custovic, Mirsad Fazlic, Sahmir Sadic, Johan Kärrholm

**Affiliations:** 1 Department of Anaesthesiology, Institute of Clinical Sciences, Sahlgrenska Academy, University of Gothenburg, Gothenburg, Sweden; 2 Department of Orthopaedics, Institute of Clinical Sciences, Sahlgrenska Academy, University of Gothenburg, Gothenburg, Sweden; 3 Swedish Joint Arthroplasty Register, Gothenburg, Sweden; 4 School of Medicine, University of Banjaluka, Banja Luka, Bosnia and Herzegovina; 5 Clinic for Orthopaedics and Traumatology, University Clinical Centre Tuzla, Tuzla, Bosnia and Herzegovina

**Keywords:** EQ-5D, hip replacement, pain

## Abstract

**Background:**

Despite the overall success of THA, between 5 and 20% report unsatisfactory results. Several factors may cause this variable outcome. 1 of them might be ethnicity which, because of its potential social impact on living conditions, may influence quality of life too. It should be studied whether patients born and being operated in their home country Bosnia and Herzegovina (BH) had similar results as immigrants being operated in Sweden (IS).

**Methods:**

Data of 280 patients were collected prospectively from questionnaires in the BH group. Patients of the IS group were eligible if both of their parents were born outside the Nordic countries, not having Swedish as their native language. Data were gained from the Swedish Arthroplasty Registry (SAR), 449 patients were included. Outcomes were pain VAS, satisfaction VAS, EQ-VAS, and the EQ-5D. Logistic and linear regression models including age, sex, diagnosis, type of fixation, surgical incision, marital status and educational level were analysed to compare those 2 groups.

**Results:**

There were considerable differences in patient demographics between the 2 groups. Before the operation, patients in the BH group reported more problems with self-care and usual activities, even after adjustment for confounding factors (*p* *<* 0.0005). Patients in the IS group reported a higher EQ-VAS and more pain VAS (*p* *<* 0.0005), the difference in the EQ-VAS was not significant after adjustment for confounding factors (*p* = 0.41). After 1 year patients in the BH group reported better scores in all dimensions of the EQ-5D (*p* ⩽ 0.005) apart from self-care. After adjustment for confounding factors, patients in the BH group were more satisfied too (*p* *<* 0.0005).

**Conclusions:**

Immigrated patients (IS group) seemed to experience less benefit from THA 1 year after the operation despite more symptoms preoperatively. There were considerable limitations affecting the results. Nevertheless, the data are a point of concern, and it is suggested to take more multidimensional care of immigrant patients.

## Background

The goal of total hip arthroplasty (THA) is to reduce pain and to improve mobility, function and quality of life. Despite the overall success of THA, up to 20% have been found to be dissatisfied after surgery.^1–3^ Various factors may cause this variable outcome. 1 of them is ethnicity which, because of its potential social impact on living conditions, will affect quality of life.^4^ Several other factors have been found to influence patient satisfaction after THA to a varying degree, including age, gender, comorbidities, preoperative diagnosis, severity of arthropathy, patient expectations, preoperative pain at rest, poorer preoperative quality of life, mental disorders and the occurrence of postoperative complications.^5–7^ Some studies have reported that there are differences in the utilisation of care due to race, without any relationship to variations in access to healthcare, income, or insurance plan.^8^ On the other hand, other studies show that ethnic disparities in THA surgery are caused by the insufficient provision of preoperative information to some immigrant patient groups. Appropriate information may reduce preoperative anxiety, which may improve surgical outcomes, shorten hospital stays and minimise any disruption of lifestyle.^9–11^ Cultural and socioeconomic background, educational level, patient expectations, pain reporting and management of pain can be more or less related to ethnicity and race.^12–15^

For decades, Sweden has welcomed immigrants from many countries around the world. During the last few years, this immigration has increased.^16^ This increase has not been accompanied by an increase in research into the way ethnic, socioeconomic, linguistic, cultural and to some extent religious issues may affect the quality of life and, as one aspect, the outcome of THA.^17^ A few studies have compared the outcome of patients born abroad with that of patients born in Sweden.^14,16–20^ Given the evidence of ethnic disparities in arthroplasty utilisation and outcomes, there is a need to identify factors potentially associated with these disparities.

In 2011, 96,467 immigrants arrived in Sweden and 26,776 of them (36%) were born in Europe outside the Nordic countries. 2173 (8.1%) of them came from Bosnia and Herzegovina. We compared the results of THA of patients born and being operated in Bosnia and Herzegovina (BH group) with patients born in Europe outside the Nordic countries, being operated in Sweden (IS group). Our hypothesis was that patients in the BH group would report superior results 1 year after the operation.

## Methods

This study was conducted at Sahlgrenska University Hospital, Gothenborg, Sweden, and 2 university hospitals in Bosnia and Herzegovina (Tuzla and Banjaluka). Data for the BH group were collected between January 2016 and December 2018 and for the IS group between 2002 and 2007.

Patients of the IS group were selected from the Swedish Arthroplasty Registry (SAR). Inclusion criteria was a complete preoperative and 1-year postoperative patient-reported outcome measure (PROM) protocol up to December 2007 Data from Statistics Sweden were used to obtain information about the country of birth, educational level and cohabitation. The simplified classification of educational level was primarily based on classes according to the International Standard Classification of Education (ISCED), as designed by UNESCO (http://www.unesco.org).

Patients for the IS group were eligible if born outside the Nordic countries with 2 parents who were also born outside the Nordic countries, not having Swedish as their native language. Patients with incomplete PROMs data preoperatively or 1 year postoperatively were excluded. 449 patients met the inclusion criteria. According to current standards in Sweden they were assumed to have visited the outpatient clinic 1–4 weeks before the operation, to be prepared for surgery. Basic demographic variables, details of the surgical procedure and implants used, details of any subsequent surgical procedure to the hip were routinely registered and reported to the SAR.

All included patients had completed a PROMs questionnaire at the pre-op and 1 year postoperatively. The questionnaire has been adapted to an internet-based touchscreen application for preoperative use in hospital clinics and it was also optionally web based. The PROM protocol comprised the EuroQol 5-dimension (EQ-5D),^18,19,21,22^ a visual analogue (VAS) for pain, an EQ-VAS, and a VAS addressing satisfaction after surgery.^23^ The orthopaedic status was documented according to the Charnley classification (A and B, C)^18,19,24^ The 3-level form that defines each dimension as no problems, some or moderate problems, or extreme problems was used. The British tariff was used to score the EQ-5D index in this population.

For the BH group the EQ-5D was translated into the Bosnian language and validated. For the first 20 patients the form was sent twice, and all the answers provided by all 20 patients were identical. All arthroplasty surgeons in Banjaluka and Tuzla were contacted to organise the data-collection. General patient information was obtained from the medical records. PROMs were assumed to be collected within 4 weeks before the operation and 1 year postoperatively. The process was supervised by a nurse at each hospital.

The nurse contacted all eligible patients about 1 month before surgery and asked for participation. Participating patients received the EQ-5D and completed it themselves, the nurse transferred their answers to an Excel file. Age, gender, diagnosis (primary or secondary osteoarthritis (OA), Charnley class (A and B, or C), education (low, middle/high), cohabitation (yes, no), type of incision (lateral, posterior) and choice of implant fixation (cemented, uncemented) were documented.

Inclusion criteria were a documented consent to participate (see below) and the completion of the PROMs questionnaires. Patients with malignancy, scheduled for 1-stage bilateral THAs, with reoperations during the study period and the first hip in patients who received their second THA were excluded.

1794 THA were performed in the 2 hospitals in Bosnia Herzogovina between 2016 and 2018. 305 of them were asked for participation, the only selection criteria was presence of a study nurse to offer and explain the study protocol to the patients. 280 (15.6%) of them agreed and had completed all forms, forming the BH group.

1591 immigrants from Europe outside the Nordic countries were operated in Swedish hospitals with a primary THA between 2002 and 2007. 449 completed the PROMs preoperatively and after 1 year (28.2%) and formed the IS group. Selection criteria for these patients were not known.

### Statistical analysis

To determine whether any of the 5 subcategories on the EQ-5D form (mobility, self-care, usual activities, pain/discomfort, anxiety/depression) was able to discriminate between patients in the IS group and in the BH group, the answers were dichotomised into either no problems or moderate/severe problems for each of the 5 subcategories. Any differences between the 2 groups were tested using a non-parametric test (chi-square, Fisher’s exact test). To evaluate any influence of covariates, we performed binary logistic regression analyses using the outcomes of no problems or moderate/severe problems for each of 5 EQ-dimensions (mobility, self-care, usual activities, pain/discomfort and anxiety/depression). The covariates included in the model were age (<60, ⩾60 years of age), gender (M/F), diagnosis (primary OA, secondary OA), Charnley class (A and B, or C), education (low, middle/high), cohabitation (yes, no), type of incision (lateral, posterior incision) and choice of implant fixation (cemented; or uncemented). The item of preoperative pain was not studied using regression analysis because, at that time point, almost all the patients reported moderate/severe problems for pain/discomfort. At the 1-year follow-up and for each of the dimensions studied (mobility, self-care, usual activities, pain/discomfort, anxiety/depression), the preoperative value for this dimension was also entered. VAS pain, VAS satisfaction and EQ-VAS were analysed using Student’s t-test, chi-square, linear and logistic regression. The deviation from normal distribution was judged to be within acceptable limits. The level of significance was set at *p* < 0.05. We used SPSS version 25.0.

### Ethics

The study was approved by the Regional Ethical Review Board in Gothenburg, Sweden (Dnr.: 271-14), the Regional Ethical Review Board in Banja Luka, Bosnia and Herzegovina (Dnr.: 116-16-8017/2016) and the Regional Ethical Review Board in Tuzla, Bosnia and Herzegovina (Dnr.: 02-09/2-4/2016). According to the Swedish Patient Data Act (2008:355), patients receive information about being registered and have every right to opt out, and informed written consent to participate was provided by every patient in the study. All investigations were performed in accordance with the declarations of Helsinki.

## Results

### Demographics

280 (38%) of the included patients belonged to the Group BH, 449 to IS. There were considerable differences in demographics: patients in the IS group were older (median 71 vs. had more primary osteoarthritis (93 vs. 19%, *p* < 0.001), belonged to a higher Charnley classification 51 vs. 20% Charnley C, *p* = *p* <0.001), more were living alone (46 vs. 11%, *p* = *p* <0.001) and had a lower education level (44 vs. 22%, *p* = *p* <0.001). There were more cemented implants (90 vs. 0%, *p* = *p* <0.001) and more lateral approach (45 vs. 25%, *p* = *p* <0.001) used in the IS group (Table 1).

**Table 1.** Patient data.

Variables	Living in Sweden, *n* (%)	Living in Bosnia and Herzegovina, *n* (%)
**Sex**		
Male	173 *(38)*	93 *(33)*
Female	276 *(62)*	187 *(67)*
**Age, mean** (95% CI)		
<60 years	81 *(18)*	124 *(44)*
⩾60 years	368 *(82)*	156 *(56)*
Median age	71 *(28-93)*	62 *(26-89)*
**Diagnosis**		
Primary OA	414 *(93)*	53 *(19)*
Secondary OA	35 *(7)*	227 *(81)*
**Charnley class**		
A+B	224 *(49)*	224 *(80)*
C	225 *(51)*	56 *(20)*
**Cohabiting**		
Yes	241 *(54)*	251 *(89)*
No	208 *(46)*	29 *(11)*
**Education** (ISCED 97)		
Low	200 *(44)*	63 *(22)*
Middle/High	249 *(56)*	218 *(78)*
**Type of fixation**		
Cemented	405 *(90)*	0 *(0)*
Uncemented	44 *(10)*	280 *(100)*
**Surgical incision**		
Lateral	203 *(45)*	71 *(25)*
Posterior	246 *(55)*	209 *(75)*

CI, confidence interval.

### Preoperative evaluation

Preoperatively, pain VAS was lower in the BH group (41 vs. 62; *p* *<* 0.0005) (Table 2). Patients in the IS group reported a higher EQ-VAS than those in the BH group (53 vs. 42; *p* *<* 0.0005), being not significant in the adjusted regression analysis (Table 4).

**Table 2.** Preoperative and 1year postoperative data of the Pain/Satisfaction and EQ-VAS, and EQ-5D questions.

	Preoperatively		*p-value*	1 year		*p-value* ^*a*^
	Mean or numbers	95% CI of mean or *percentages*		Mean or numbers	95% CI of mean or *percentages*	
**Pain VAS**						
Living in Sweden (IS)	62	61-64	*<0.0005*	19	17-21	*<0.0005*
Living in Bosnia and Herzegovina (BH)	41	40-43		15	14-17	
**Satisfaction VAS**						
Living in Sweden (IS)				20	18-22	*<0.0005*
Living in Bosnia/Herzegovina (BH)				13	12-13	
**EQ-VAS**						
Living in Sweden (IS)	53	51-55	*<0.0005*	71	69-73	*<0.0005*
Living in Bosnia and Herzegovina (BH)	42	40-44		76	74-78	
**EQ-5D questions** *no problems/moderate or severe problems*						
***Mobility***						
Living in Sweden (IS)	19/430	*4/96*	*0.5*	242/207	*54/46*	*<0.0005*
Living in Bosnia and Herzegovina (BH)	11/269	*4/96*		254/26	*91/9*	
***Self-care***						
Living in Sweden (IS)	309/140	*69/31*	*<0.0005*	378/71	*84/16*	*0.8*
Living in Bosnia and Herzegovina (BH)	85/195	*30/70*		234/46	*84/16*	
***Usual activities***						
Living in Sweden (IS)	162/287	*36/64*	*<0.0005*	296/153	*66/34*	*<0.0005*
Living in Bosnia and Herzegovina (BH)	40/240	*14/86*		245/35	*87.5/12.5*	
***Pain/discomfort***						
Living in Sweden (IS)	5/444	*1/99*	*0.05*	167/282	*37/63*	*<0.0005*
Living in Bosnia and Herzegovina (BH)	9/271	*3/97*		238/42	*85/15*	
***Anxiety/depression***				H		
Living in Sweden (IS)	195/254	*43/57*	*0.7*	293/156	*65/35*	*0.001*
Living in Bosnia and Herzegovina (BH)	126/154	*45/55*		216/65	*77/23*	

CI, confidence interval; VAS, visual analogue scale.

a *t*-test or chi-square.

A higher percentage of patients in the BH group reported moderate to severe problems with self-care and usual activities (EQ-5D) (Table 2). This was also found in the unadjusted and adjusted logistic regression analyses (Table 3).

**Table 3.** Results of logistic regression analyses for the 5 EQ-5D questions.

	Preoperatively		*p-value*	1 year		*p-value*
	OR	95% CI of mean or *percentages*		OR	95% CI of mean or *percentages*	
**EQ-5D questions** *no problems/moderate or severe problems*						
***Mobility***						
Unadjusted	0.9	*0.4–2.0*	*0.8*	8.36	*5.4–13.0*	*<0.0005*
Adjusted	0.4	*0.1–1.4*	*0.15*	5.40	*3.0 -9.8*	*<0.0005*
***Self-care***						
Unadjusted	0.2	*0.1–0.3*	*<0.0005*	0.96	*0.6–1.4*	*0.8*
Adjusted	0.2	*0.1–0.3*	*<0.0005*	1.53	*0.8–3.0*	*0.2*
***Usual activities***						
Unadjusted	0.3	*0.2–0.4*	*<0.0005*	3.62	*2.4–5.4*	*<0.0005*
Adjusted	0.3	*0.2–0.6*	*<0.0005*	3.52	*2.0–6.2*	*<0.0005*
***Pain/discomfort***						
Unadjusted	-^*a*^			9.57	*6.6–14.0*	*<0.0005*
Adjusted	-			7.36	*4.8–11.2*	*<0.0005*
***Anxiety/Depression***						
Unadjusted	1.1	*0.8–1.4*	*0.7*	1.76	*1.3–2.5*	*0.001*
Adjusted	1.1	*0.7–1.8*	*0.7*	2.14	*1.3–3.6*	*0.005*

CI, confidence interval.

Values >1 indicate that patients living in Sweden more frequently report moderate or severe problems.

a No analysis because almost all patients ticked same level of pain (no variation).

### Postoperative evaluation

1 year after the operation, the VAS pain was lower, the satisfaction-VAS and EQ-VAS higher in the BH group (Tables 2 and 3). Patients in the BH group had better results in all dimensions in the EQ-5D, apart from self-care. After adjustment for confounding, the results changed only marginally (Tables 2–4).

**Table 4.** Analyses of pain: satisfaction and EQ-VAS in general linear models.

	Preoperatively		*p-*value	1 year		*p*-value
	B	*95% CI*		B	*95% CI*	
**Pain VAS**						
Unadjusted	20.7	*18.1–23.3*	*<0.0005*	3.2	*0.6–5.9*	*0.18*
Adjusted^*a*^	15.74	*12.0–19.5*	*<0.0005*	2.4	*−1.7–6.5*	*0.25*
**Satisfaction VAS**						
Unadjusted	-			7.3	*4.6–10.0*	*<0.0005*
Adjusted^*a*^	-			8.7	*4.5–12.9*	*<0.0005*
**EQ-VAS**						
Unadjusted	10.9	*7.8–13.9*	*<0.0005*	−5.2	*−8.2–−2.3*	*0.001*
Adjusted^*a*^	2.0	*−2.8–6.7*	*0.41*	−5.2	*−9.7– 0.7*	*0.25*

VAS, visual analogue scale.

a Adjusted for age, gender, diagnosis, Charnley class, incision, cohabitation and for pain VAS and EQ-VAS at 1 year also preoperative value.

## Discussion

Few studies compare outcomes between patient groups with similar ethnicity living in their country of birth or in a foreign country to which they have emigrated. Some have focused on PROMs of various immigrant groups within a country using the native population as reference. In a recent study, we examined immigrants in Sweden divided into 3 groups, depending on the region of birth, and compared the PROMs 1 year after THA with those of patients born in Sweden.^18,19^ In these studies, immigrants^15,18,19^ reported more problems in the dimension of self-care and usual activities and had a tendency towards a higher level of anxiety/depression the longer the distance to their country of birth. Immigrants born in Europe outside the Nordic countries ran a higher risk of reoperation than immigrants born outside Sweden. They reported more frequently being poorly informed as compared to the native Swedish population.^15,18,19^

For the presented study European countries outside the Nordic region were grouped together as no further detailed information was available. Our study confirms previous findings that immigrants run an increased risk of inferior results after THA. There are numerous possible reasons, such as a history of living in areas of conflict and a traumatic escape from these areas, loss of relatives and friends, loss of previous social status and, in some cases, a deteriorating economy. Even though we did not know the specific home country in the IS group, our observations support the hypothesis that the inferior results in the IS group might be related to their immigrant status.

Oldsberg et al.^25^ studied PROMs before and after THA in different Swedish counties. The found variations could not be explained by differences in the preoperative data after adjustment for patient demographics, including socioeconomic variations. They thought that local variations in outcome could be due to differences in indications for surgery and a lack of standardised care. This theory was partly supported by Judge et al.^26^ who noted that the incidence of THA in various regions of England was influenced by sociodemographic factors. In our study indications for surgery might have varied between the 2 groups of patients too and could have been a source of bias.

Our study has further considerable limitations. Firstly, the 2 groups differed significantly in most of the assessed demographic and surgical data. The main reason for these differences can be explained. In Sweden almost all health care is publicly financed, whereas in Bosnia and Herzegovina the patients pay themselves for elective hip surgery. This difference will most probably have profound effects on patient selection. Further, the surgical tradition concerning choice of implants and surgical approaches differs between countries. Another limitation with our study is that general comorbidities were not assessed. Despite that we tried to compensate for these discrepancies in the regression analysis, we still think that that the variation within each group of patients was not big enough to avoid a remaining influence on the final results.

As far as we are aware, the EQ-5D form has not previously been tested in a refugee population, which is a weakness of our study and affects the validity of the results too.^27^ We could not penetrate the amount of refugees in the IS group but there were no refugees included in the BH group. Previous studies have revealed some inconsistencies when using the EQ-5D cross-culturally. Thus our results are time and context bound and at best only apply to the current study population. It remains unknown whether these forms are transferable to other refugee groups with a different national background.^28^

Furthermore, the influence of environmental factors could not be controlled but it can be assumed that they differed between the 2 countries too.

The PROMs were collected by different means (registry vs. explanation and distribution by a study nurse) and the data for the IS group were generated >10 years before the BH group, which might have affected the results.

In the observation period for the IS group many hospitals had not yet adopted the PROM programme, thus the observation period was long and many patients were not integrated in the progamme. When collecting data for the BH group, many patients were excluded due to a lack of resources to explain the study and PROMs. In both groups complete forms were only available in a small fraction of all operated patients and a selection bias cannot be excluded. The reasons for exclusion in both groups were multiple and different, thus the effect of the high number of drop-outs on the results remains very speculative.

In the BH group 3 surgeons on each of the 2 hospitals did all operations, being specialists in their field. In the IS group all arthroplasty surgeons could have done the operations including those under education, thus there was more variation in the surgical experience. But as a rule, surgeons under education are guided by an experienced senior colleague. In a recently performed study from the SAR variations in surgical experience did not or only marginally influence PROMs after 1 year (Jolbäck et al.^29^). Thus it seems little probable that variations in surgical experience explain the found difference in PROMs.

An explanation for our findings might be differences in the healthcare system between Sweden and Bosnia and Herzogovina and cultural differences, like the participation of the family in the care of patients. A previous report pointed out that in Southern Europe like in Greece or Spain and in Bosnia Herzogovina too, there is more involvement of the family in care than in the northern countries.^30–32^ Families, with all their aspects, often take the role of an informal caregiver, being imposed by cultural norms and promoted by the society.^33^ Hospitals and hospital rooms may not have differed between patients being treated in BH or IS groups, but it is very probable that the immigrant patients in the IS group experienced more problems related to the cultural identity compared to the BH patients, being treated at home.

Language barriers and communication problems can negatively affect the outcome of a surgical procedures.^15,17^ In the present study, patients in the BH group communicated in their mother language, most had surgery in the city in which they were born, had regular support and family visits and had the same ethnic and cultural background as their caregivers. For patients in the IS group, the situation was more or less the opposite.

Several studies found that quality of life as assessed with HRQoL instruments and self-perceived health status increased with improved socioeconomic status and education.^34–36^ In our study, the income of the patients could not be analysed. The educational level of patients in the BH group was higher but this has to be seen in the light of locally available diplomas or certificates of educational degrees that are easily purchased on the black market. Thus, it is questionable whether this is an explanation for the found results.

## Conclusion

The immigrants in the IS group reported fewer problems with health and usual activities before surgery than in the BH group, being operated in their home country. 1 year after THA, patients in the BH group had a higher degree of improvement and of satisfaction than the IS group. A more comfortable, socially more secure home environment might be 1 explanation. Many factors such as the healthcare system itself, reimbursement for THA and limited intercultural comparability of PROMs could not be ruled out and make comparisons in a more scientific way difficult. But since the number of immigrants is increasing in most European countries, further studies are needed to evaluate and optimise the healthcare situation for these patients.
